# Retraction: Cytotoxic effects of *Lavandula angustifolia* seed extracts on the viability of Huh-7 and Chang liver cells

**DOI:** 10.1371/journal.pone.0275289

**Published:** 2022-09-30

**Authors:** The PLOS ONE Editors

This article [1] was identified as one of a series of submissions for which we have concerns about authorship, competing interests, and peer review. Based on the outcome of our assessment, the *PLOS ONE* Editors concluded that this article [1] was published on the basis of a compromised peer review process.

The following issues were also noted in our internal assessment:

- Within each figure panel, the reported standard errors are highly similar across experimental conditions. This calls into question the reliability of the reported results.
- Contrary to the article’s Data Availability Statement, the raw individual-level data used to generate the graphs were not provided with the article as is required by the *PLOS ONE* Data Availability Policy.
- The Chang liver cell line has been reported to be contaminated and to be a HeLa derivative, not a cell line of hepatic origin [2].

The corresponding author acknowledged that standard errors are presented incorrectly, and they stated that the raw individual-level data underlying the graphs are available.

In light of the above concerns, particularly those involving the peer review process and the reliability of the reported results, the *PLOS ONE* Editors retract this article. We regret that the issues were not addressed prior to the article’s publication.

The author did not agree with the retraction.
